# Strategies for the Design and Construction of Nature-Inspired & Living Laboratory (NILL 1.0)^TM^ Buildings

**DOI:** 10.3390/biomimetics9070441

**Published:** 2024-07-18

**Authors:** Mariam AlAli, Salwa Beheiry, Serter Atabay

**Affiliations:** 1 Engineering Systems Management, American University of Sharjah, Sharjah 26666, United Arab Emirates; 2 Department of Civil Engineering, American University of Sharjah, Sharjah 26666, United Arab Emirates

**Keywords:** nature-inspired, living, laboratory, biophilic, construction, strategies, buildings, biomimicry, green, sustainable

## Abstract

This article explores the growing prominence of nature-inspired design philosophies in the context of sustainability and human well-being within the built environment and focuses on their application within laboratory buildings. Biomimicry and biophilic design are highlighted as key nature-inspired design approaches, with biomimicry drawing inspiration from nature for innovations and biophilic design promoting human health through enhancing the connection with the surrounding natural elements. This paper further discusses living building strategy as an emerging method for creating dynamic and adaptable spaces by prioritizing user experience through co-creation and focusing on sustainable and regenerative structures. The potential of integrating these approaches is emphasized using laboratory buildings as an example, with nature-inspired and living laboratories serving as models for future built environments that promote both environmental responsibility and a positive human experience. Accordingly, this work aims to investigate the design and construction of laboratory buildings based on nature-inspired design strategies and the living building concept. Moreover, the paper discusses the application of biomimicry and living building concepts within laboratory buildings as a novel contribution to the body of knowledge, and concludes by proposing the Nature-inspired & Living Laboratory (NILL 1.0)^TM^ Building Assessment index to serve as a guideline for the design and construction of laboratory buildings using nature as an inspiration and the analogy of human body systems.

## 1. Introduction

Nature-inspired design is becoming more popular in a world where sustainability and wellbeing are increasingly prioritized. Biomimicry and biophilic design are two methods that fall under the umbrella of nature-inspired design [1]. By directly imitating natural forms, processes, and ecosystems, biomimicry attempts to address problems facing humanity and produces innovations in construction methods, building materials, and energy systems [2,3]. Additionally, biophilic design, much like green and sustainable design that emerged in the 1970s, aims to improve human comfort, productivity, and health by bringing natural elements into the built environment [4]. These design philosophies offer numerous advantages by utilizing the power of nature, encouraging environmental responsibility as well as an enhanced human experience in the built environment [5].

Furthermore, biophilic design has been identified by Kellert and Wilson in 1993 as “the deliberate attempt to translate an understanding of the inherent human affinity to affiliate with natural systems and processes”, known as biophilia [6]. This definition was further developed by Kellert in 2011, who emphasized that “the positive experience of natural systems and processes in our buildings and constructed landscapes remains critical to human performance and well-being” [7]. On the other hand, biomimicry, a promising research field providing possible nature-inspired solutions for design problems, has been coined as a term by Janine Benyus in 1997 through merging the Greek words “bios”, meaning “life”, and “mimesis”, meaning imitation [8]. Over the span of 3.8 billion years, natural processes have developed technologies comparable to, or surpassing, those created by humans, using sustainable and efficient methods [9]. Therefore, biomimicry, an interdisciplinary scientific field, holds promise for delivering sustainable solutions through collaboration among biologists, physicists, chemists, engineers, and architects [10]. 

Moreover, creative methods for designing dynamic, flexible spaces are emerging as the built environment changes to meet the demands of the 21st century. Two examples of these developments are living laboratories and living building techniques. By encouraging co-creation and in-the-moment testing of design features, living labs put the user experience first [11]. With the help of this user-centered approach, researchers, users, and designers can work together to create inventive and useful spaces. On the other hand, living building strategies concentrate on developing structures that are sustainable, regenerative, and functional [12]. These tactics seek to reduce their negative effects on the environment, maximize resource efficiency, and even improve the local ecosystem [13]. Through the integration of these approaches, living laboratories can be designed to serve as models for innovative design as well as showcases for sustainable and user-centric built environments.

Accordingly, this paper aims to explore the approaches/practices employed in the design and construction of laboratory buildings based on the concepts of Living Lab and Living Building and nature-inspired design through biophilic design and biomimicry. The aim is achieved through setting multiple objectives, which include the review of peer-reviewed journals published through the international database SCOPUS from 2000 until 2024, highlighting the major research contributions within the area of the topic of interest, and, finally, providing a set of strategies to design and construct Nature-inspired & Living Laboratory (NILL) Buildings through the Nature-inspired Living Laboratory (NILL 1.0)^TM^ Assessment Tool.

## 2. Materials and Methods

### 2.1. Data Collection

This article explored the application of nature-inspired strategies, including biomimicry and biophilic design, as well as the living lab approach and living building concept within the context of laboratory buildings. The data were primarily collected from a large assortment of scholarly articles acquired from SCOPUS database and Google Scholar. The examined publications covered the time period between 2000 and 2024 and used the following code for SCOPUS search: (“Biomimicry” OR “Living Building Challenge” OR “LBC” OR “biophilia” OR “biophilic design” OR “sustainable construction”) AND (“lab*” AND “building”). The reviewed publications were selected based on the following criteria: range of publication between years 2000 and 2024, relevance of title and abstract to the addressed topic, and the content being relevant to architecture, construction, and engineering aspects of laboratory buildings, while ensuring that the publication is in the final stage and written in the English language. Moreover, the exclusion criterion strictly omitted any publication not belonging to a SCOPUS-indexed scholar. However, due to the lack of scholarly articles discussing the application of the previously mentioned concepts within laboratory buildings, additional resources, such as conference papers and official websites, were reviewed through search queries in Google Scholar.

### 2.2. Data Processing and Text Mining

Titles and abstracts were scanned for relevance to the selected topics of biomimicry, biophilic design, living lab approach, and living building within laboratory buildings in order to facilitate text mining and the review process of the selected papers. Papers were then categorized according to their respective fields. Using Microsoft Excel (Microsoft Office Home and Student 2019 version 2304, Redmond, WA, USA), themes and key ideas were taken from the papers and entered into tables. The main themes that were addressed while examining the articles were applications or case studies concerning biomimicry, biophilic design, living laboratory, living building, and sustainable building. Those themes were used to categorize the articles that are further discussed and summarized in the Section 3. Where data from the articles that appeared in SCOPUS were not enough to proceed with the next phase of the methodology, additional resources such as Google Scholar were used to capture relevant articles using the same keywords mentioned in Section 2.1.

### 2.3. Index Creation

The reviewed literature was assessed for the extraction of relevant features/indicators that can be compiled within an index to assess laboratory buildings by combining key features inspired by biomimicry, biophilic design, living building, and sustainable features. The indicators were then categorized into relevant groups/constructs under one common theme that collectively combines similar aspects. The main approach of designing the index was based on the analogy of the human body, which consists of different systems performing specific functions. Accordingly, each system was applied within the context of a laboratory building based on its primary function and how it can be applied or utilized for inspiration to create a similar system in a laboratory building. Therefore, each system is considered as a separate category or “construct” within the index. Furthermore, the indicators of each category/system were chosen based on their suitability to achieve the overall purpose of the category/system. Overall, this conceptual guideline is proposed as a first stage of multi-stage research that will include a future validation for the entire index and all its attributes through subject matter experts from the industry and subjected to further quantitative analysis and possible future publication.

## 3. Results

### 3.1. SCOPUS Search

**Figure 1:**
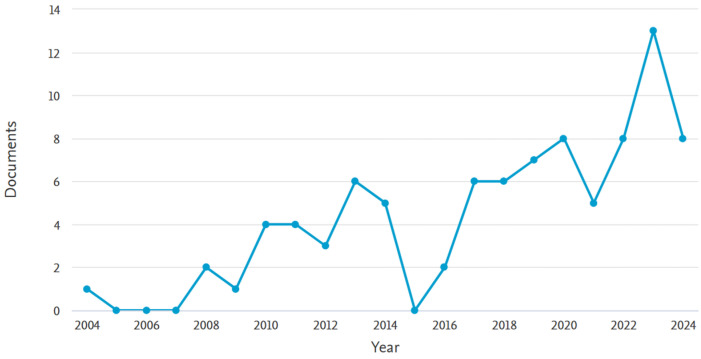
Number of annual publications around the topic of interest during the period of 2000–2024.

The results of the initial search through SCOPUS database using the keywords mentioned in the methodology showed a low number of publications during the period of 2000 until 2024, ranging between 1 and 13 publications per year, as shown in Figure 1. However, the topic under investigation is still relatively new and not thoroughly investigated. Overall, the accumulative total of the SCOPUS-indexed articles was 89 articles, of which a selection is examined and further discussed in this section.

**Figure 2:**
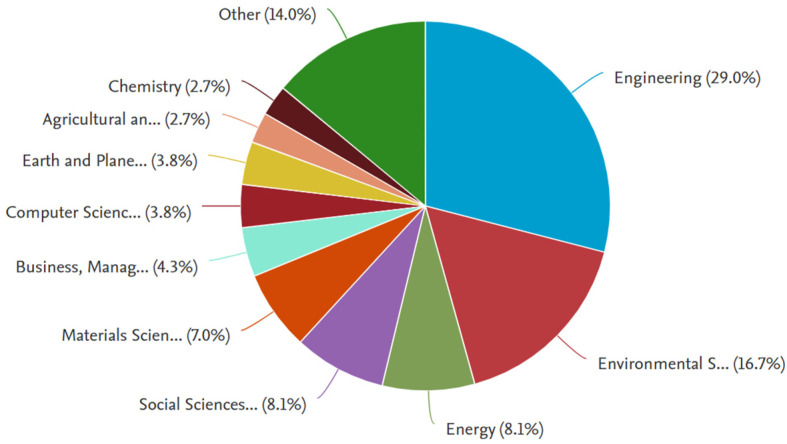
Research interest from different fields concerning the topic of interest during the period of 2000–2024.

Further analysis into the publications found on SCOPUS suggests that the topic under investigation has received a relatively equal percentage of interest amongst researchers in the fields of Energy and Social Sciences, with the highest interest being in Engineering, followed by Environmental Sciences, as shown in Figure 2, which further supports the notion that there is a need to consolidate the acquired knowledge in all three fields in a comprehensive approach to effectively address the topic of interest.

### 3.2. VOS Viewer Analysis

**Figure 3:**
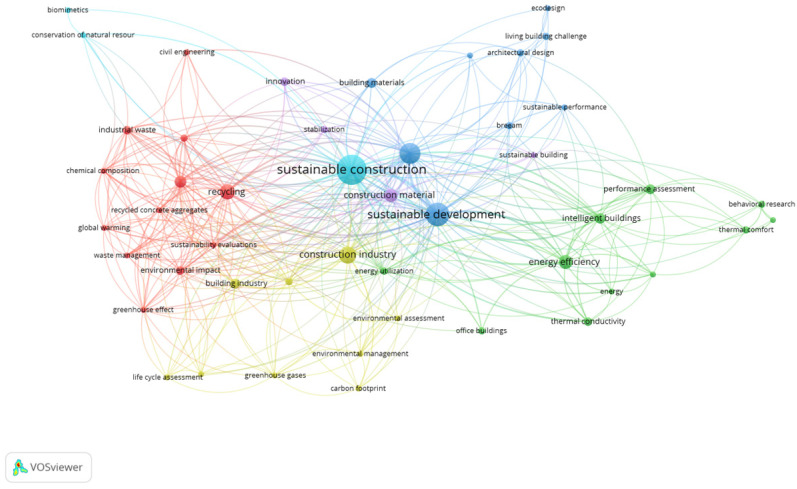
Author keywords mentioned at least two times within the publications during the period of 2000–2024.

Additional analysis into the trending keywords using VOS Viewer relied on the criterion of selecting keywords that appeared two times or more within the 89 selected articles. As a result, 21 publications showed several links between multiple terminologies emerging from the topic of interest, such as biophilia, living building challenge, architectural design, sustainability, sustainable construction, living buildings, energy efficiency, thermal comfort, and biophilic design, amongst others, as shown in Figure 3. Accordingly, this analysis further supports the notion that the consolidation of biomimicry, biophilic design, living building, and sustainable features has started to capture the interest of researchers, with potential for further enhancements and developments in the future.

### 3.3. Summary of Articles Findings

Further examination of the extracted 21 articles through evaluating the abstract content, purpose, methodology, and results reduced the number of articles to 12 articles, with their main findings summarized in Table 1.

Focusing on the importance of biomimicry education in sustainability [15], Yeter et al. conducted workshops in Singapore to explore how students from two distinct groups—local high school students and undergraduate engineering students from the United States—conceptualized biomimicry approaches. The study concluded that students struggled with the top-down method, which involves identifying human problems and seeking solutions from nature. This highlights the need for curriculum development that strengthens students’ ability to identify the unique principles that make natural objects function effectively and fosters interdisciplinary knowledge for a more comprehensive grasp of biomimicry.

As for the application of biophilic design, the SCOPUS search revealed a study by Jiang et al. that investigated the impact of windows on occupants in buildings [16], finding that having a window led to increased comfort and tolerance of thermal conditions (potentially saving energy), reduced stress and fatigue (based on physiological measurements), and offered energy-saving potential through daylight, though it did not significantly affect perceived lighting, as users were satisfied with both levels of natural and artificial lighting. Overall, integrating windows as a biophilic design feature to enhance user experience and comfort level was evaluated and showed potential for further exploration and consideration in building design.

Concerning the use of living building materials in the construction of buildings, a study by Crawford et al. explored the potential of microalgae, a type of microscopic organism, as a sustainable building material [17]. Researchers investigated the use of 3D-printed ceramic structures embedded with microalgae to create “living” building components. The study evaluated how different designs affected the survival and growth of the algae. The findings suggest that integrating these micro-ecologies into buildings could contribute to sustainability efforts and mitigate anthropogenic carbon dioxide emissions.

The use of living building components, such as living walls, has also been discussed by Assem and Hassan [14]. This study investigated the use of living walls, plant installations integrated into walls, to improve employee well-being and combat sick building syndrome in workplaces. Researchers employed a parametric design approach to optimize the design of these living walls, considering factors like aesthetics, user interaction, and functionality within the workspace. The results, though not explicitly detailed, likely demonstrate the effectiveness of this approach in creating visually appealing living walls that enhance user experience and create unique ambiances suitable for various workplace activities.

The application of the Living Building Challenge (LBC) has also been discussed in a previous study by Cianfrani et al. [22]. This study examined the R.W. Kern Center, Hampshire College’s first new building in 40 years, designed with a focus on forward-thinking sustainability. The multi-purpose facility aims to be self-sufficient in energy generation, water management, and waste processing. It prioritizes nontoxic materials, local sourcing, and biophilic design principles to promote human well-being and natural beauty. Beyond functionality, the building serves as a gateway to the campus, attracting prospective students and fostering a sense of community. The R.W. Kern Center achieved Living Building Challenge certification in 2018, demonstrating its commitment to a holistic approach to sustainability.

Energy consumption patterns was another theme discussed within the reported studies. One study by Yeter et al. investigated the effectiveness of three energy-saving initiatives in a university office/laboratory building designed with sustainable features, such as natural ventilation in an open-plan office area and biomass to meet heating requirements, at the University of Cambridge [24]. The building employed voluntary design frameworks (BREEAM) and a bespoke post-construction strategy (Cambridge Work Plan) alongside mandatory EU reporting requirements. Eventually, the study revealed a significant discrepancy between the building’s actual energy consumption (140% higher than estimated needs) and its design projections. Furthermore, the three initiatives implemented fell short in actively reducing this energy performance gap. As such, the study recommended alternative approaches that prioritize monitoring operational performance and ensure realistic energy estimations during the design stages, including building energy management strategies focused on operational performance, such as the Living Building Challenge [25]. As such, LBC can drive buildings to achieve net-zero energy consumption using renewable sources, thus integrating biomimicry to mimic nature’s efficiency and resilience. It can also promote biophilic design by enhancing occupant well-being through natural elements like light and vegetation, while reducing energy needs. Thus, LBC can support harmonizing environmental responsibility with human comfort and health. Other suggested tools for utilization included the Chartered Institution of Building Services Engineers’ Technical Memorandum 54, a technical guideline that aids building professionals during building design to create more accurate energy models by transforming low-energy designs into buildings that meet designated energy targets and offering precise instructions for thoroughly and accurately assessing operational energy consumption during the design phase [26].

Overall, the result of analyzing the SCOPUS-indexed articles concerning the topic of interest revealed a general lack of application of biomimicry, biophilic design, and living building strategies within laboratory buildings. Further suggestions are made by the authors on the applicability of those concepts and detailed in the Discussion section.

## 4. Discussion

### 4.1. SCOPUS-Indexed Publications

Building on the findings in the previously mentioned study of biomimicry application in education [15], the study’s outcomes can be applied to bridge this gap by emphasizing the importance of understanding nature’s “unique principles”. This could involve studying phenomena like termite mounds for inspiration in passive cooling systems or mimicking spider silk’s strength-to-weight ratio for lightweight structures. Additionally, fostering interdisciplinary knowledge by combining biology, engineering, and architecture can lead to broader biomimicry inspiration. By addressing these knowledge gaps, professionals can be equipped with the skills to translate biomimicry into practical applications for laboratories. This can ultimately lead to the design of more sustainable, adaptable, and user-centric lab environments that promote scientific progress.

Regarding the application of biophilic design in the built environment that was previously discussed [16], the reported findings support the notion of using biophilic design principles, which incorporate elements of nature like natural light and views to enhance occupant well-being and sustainability in laboratory buildings. To explain, incorporating windows with natural views can create a more comfortable and healthier workplace for lab personnel, as natural light and views can potentially lead to increased comfort in cooler temperatures and reducing energy consumption for cooling. Additionally, biophilic design principles promote a connection with nature, which has been shown to decrease stress and fatigue in occupants, leading to increased productivity and better job performance. Thus, windows can serve as a valuable design feature to improve the access to nature through the building envelope, while other alternatives may also provide a similar experience when windows are not present in the building, such as a roof glass ceiling. By integrating these elements, architects and designers can create lab environments that prioritize both human health and sustainability.

Concerning the use of living building materials in the construction of buildings using microalgae, as discussed previously [17], this holds promise for laboratory facilities. To illustrate, laboratories often require specific environmental conditions, and these living materials could potentially help regulate temperature or air quality within the space. Additionally, the controlled environment of a laboratory would be ideal for studying the interaction between microalgae and different materials. Researchers could leverage this setting to further explore the potential benefits and limitations of these living materials for broader use in architecture.

The concept of living walls in building setups, as previously discussed [14], has potential for application in laboratory facilities. To illustrate, laboratories can often be sterile and lack natural elements. Therefore, living walls could introduce a connection to nature, potentially improving employee well-being and reducing stress, which can be crucial for scientific research. Additionally, the study’s focus on user interaction with the living wall could be particularly relevant in a lab setting. Interactive elements could provide employees with opportunities to engage with nature during breaks or stressful moments, further promoting well-being.

Moreover, the LBC certification may be assessed for suitability to apply in laboratory buildings, taking buildings such as R.W. Kern Center as an example to follow [22]. To explain, the sustainable design principles employed at the R.W. Kern Center hold promise for application in laboratory buildings, since laboratories often require significant energy and water resources. Therefore, implementing similar self-sufficiency measures could minimize environmental impact. Additionally, focusing on nontoxic materials and biophilic design could promote the health and well-being of laboratory personnel, who are considered a crucial factor in scientific research. By adopting these principles, laboratories can contribute to a more sustainable future while creating healthy and inspiring work environments for scientific discovery.

Additionally, the findings of the study on energy consumption at the University of Cambridge [24] hold significant value for laboratory buildings, which are known for high energy consumption. The identified shortcomings of focusing solely on design-stage initiatives highlight the need for a multi-pronged approach. Laboratories can benefit from incorporating operational performance monitoring into their energy management strategies. Additionally, collaborating with design professionals who utilize tools like the Chartered Institution of Building Services Engineers’ Technical Memorandum 54 can ensure the creation of realistic energy models at the design stage. By implementing these recommendations, laboratory buildings can bridge the energy performance gap and achieve true sustainability goals.

Overall, the potential of applying nature-inspired design strategies and the living building concept within laboratory buildings is still a relatively new field, with significant potential for further work and advancements. The authors discuss some strategies detailed in the following subsections.

### 4.2. Commandments of Biomimicry and Laboratory Buildings

Pioneering advocate for biomimicry, Janine Benyus, established the core principles, often referred to as “commandments”, that provide a framework for applying biomimicry to problem solving across various disciplines [8]. These principles hold significant influence within the built environment, shaping sustainable and innovative design approaches. By encouraging the study of nature’s models, ecosystems, and processes as a source of inspiration, biomimicry translates into real-world applications such as mimicking natural ventilation systems for passive cooling or emulating spider silk for lightweight building materials. Furthermore, these principles advocate for using nature as a benchmark for measuring the environmental impact and as a mentor for fostering adaptable designs. Buildings designed with biomimicry in mind can minimize their environmental footprint by considering energy use and resource consumption throughout their lifecycle. Additionally, these principles can inspire the creation of structures that evolve over time, similar to how organisms adapt to their environment. Finally, there is an emphasis on the importance of respecting the interconnectedness of nature. This translates to buildings designed with locally sourced materials and a consideration for the impact on the surrounding ecosystem. By adhering to these principles, architects and engineers can create buildings that are more sustainable, resilient, and user-centric, ultimately promoting a built environment that thrives in harmony with the natural world.

As the current literature does not discuss the application of Benyus’s commandments of biomimicry to laboratory buildings, the authors suggest the following strategies (Table 2), where each commandment is applied within the context of a laboratory building to serve as a general guidance for designers, contractors, laboratory managers, or laboratory building users/occupants.

### 4.3. Biomimicry Life Principles and Laboratory Buildings

The organization Biomimicry 3.8 acts as a champion for the field of biomimicry, drawing inspiration from the vast library of biological strategies gleaned from Earth’s 3.8-billion-year evolutionary history [27]. This knowledge base informs the development of the Biomimicry Life Principles, which constitute a core element of Biomimicry 3.8’s methodology. These principles function as a blueprint for sustainable and efficient design, guiding designers and engineers to emulate nature’s time-tested patterns and processes. Furthermore, Biomimicry 3.8 offers tools such as the DesignLens: Life’s Principles to facilitate the application of these principles across a wide range of design challenges [28]. In essence, the Biomimicry Life Principles serve as the foundational pillar upon which Biomimicry 3.8 constructs its approach. By harnessing nature’s wisdom through these principles, Biomimicry 3.8 fosters the development of ecologically sustainable and innovative solutions. This ensures that human creations are well adapted to the Earth’s ecosystem and contribute positively to it.

As the current literature shows lack of publications discussing the application of Biomimicry Life Principles in laboratory buildings, the authors present several strategies (Table 3) where each principle is translated into a practical application during the design, construction, and operation of a laboratory facility.

### 4.4. Living Building Challenge (LBC) and Laboratory Buildings

The Living Building Challenge Imperatives offer a thorough framework for creating living laboratories inspired by nature that aim for a regenerative impact rather than just sustainability [29]. These Seven Imperatives [30], which are divided into seven interconnected petals (Place, Water, Energy, Health & Happiness, Materials, Equity, and Beauty), have a major impact on all phases of a laboratory’s lifecycle.

To further illustrate this possible application, the “Place” petal has “Imperative 01: Site Ecology” that necessitates a detailed examination of the surrounding environment, which may have an impact on the design stage by prescribing a building footprint that reduces disturbance and blends in with the natural features already in place. Then, using biomimicry features (motivated by the “Beauty” petal) like naturally occurring ventilation systems modelled after animal respiratory systems can be considered, which lessens the need for mechanical equipment and advances the “Energy” petal. Moreover, to lessen its impact on the environment, construction (the “Materials” petal) may use recycled or readily renewable materials that can be found locally (Imperative 05: Biobased & Recycled Content). Rainwater harvesting systems in line with the “Water” petal’s “Imperative 09: Water Capture & Treatment” can considerably lessen dependency on municipal supplies during operation and management. Meanwhile, occupant well-being tactics from the “Health & Happiness” petal, such as plenty of natural light and better indoor air quality, can boost user satisfaction and productivity. These are just a few instances that demonstrate how Living Building Challenge Imperatives, when carefully implemented, can turn labs into functional, naturally inspired living ecosystems that also improve the surrounding area and the health of their occupants. As such, the authors propose the following tactics (Table 4) where each imperative of the LBC’s petals can be applied in a laboratory building, along with the predicted implications.

### 4.5. Comparative Overview of Biomimicry and LBC in Laboratory Buildings

A significant alignment exists between the philosophies of biomimicry and the Living Building Challenge (LBC), both advocating for a built environment that fosters harmony with nature. Biomimicry draws inspiration from biological systems, encouraging the design of buildings that emulate nature’s efficiency and resilience in areas like resource management and structural integrity. The LBC, on the other hand, establishes a rigorous framework for sustainable construction, pushing for buildings to function as self-sufficient ecosystems with minimal environmental impact. This synergy between biomimicry and the LBC presents intriguing possibilities for the future of architecture, particularly within the domain of laboratory buildings. By integrating biomimicry principles into LBC design strategies, laboratories have the potential to evolve beyond their role as purely functional research spaces, transforming into models of environmental responsibility. One can envision buildings that utilize natural ventilation strategies inspired by termite mounds, or self-cleaning facades mimicking the lotus leaf. These biomimetic elements, when coupled with the LBC’s emphasis on renewable energy and water conservation, could lead to the creation of highly sustainable and functional laboratory buildings. This convergence of biomimicry and the LBC holds the potential to transform the built environment, fostering a future where human innovation coexists seamlessly with a healthy and thriving natural world. A detailed comparison between biomimicry and LBC is further discussed in Table 5.

### 4.6. Nature-Inspired and Living Laboratory (NILL 1.0)^TM^ Building Assessment Index

The authors propose a novel index system for the assessment of laboratory buildings based on nature-inspired strategies and the living building concept. The index is based on a novel approach for enhancing laboratory buildings by leveraging the combined potential of biomimicry, biophilic design, and Living Building Challenge (LBC) principles, all evaluated through the NILL assessment index. The NILL index introduces the concept of a Living Laboratory—a platform that fosters curiosity, sparks innovation, and actively contributes to the advancement of nature-inspired solutions. By integrating biomimicry and biophilic design as core elements, the NILL index would create a framework for sustainable design strategies within the Living Laboratory. This dynamic environment would engage users, researchers, and the public through multisensory experiences, nurturing a collaborative learning culture. It is important to note that the current index system is a qualitative guideline, which will be further developed and quantitatively validated in future work.

The core concept of the NILL index lies in the synergy between biomimicry, biophilic design, and the interconnectedness of the human body’s systems. The LBC framework, which emphasizes buildings functioning in harmony with nature, aligns beautifully with this biological principle. The NILL index takes this analogy beyond function. The human body thrives due to a network of integrated systems—respiratory, circulatory, nervous, etc.—working together seamlessly. Similarly, the NILL index encourages laboratories to operate as a cohesive unit, with each design element (ventilation, lighting, and water management) contributing to a holistic and sustainable environment that reflects biomimicry and biophilic design principles. By integrating these core elements with LBC principles, the NILL index and the living laboratory concept can transform laboratory buildings into models of sustainable design, user-centric innovation, and a newfound understanding of the built environment’s role in supporting human health and well-being. Table 6 shows the overlay between the main themes of the NILL index and the connection with the LBC petals and the human body systems.

Furthermore, the proposed NILL index provides a connection between each of its themes, the parallel LBC petal and the relevant Biomimicry Life Principles (Table 7) to showcase the essence of the index and its main purpose of mimicking nature’s best and implementing nature’s best practices and unique strategies in achieving the highest level of performance for a laboratory building.

To further explain the respective themes within the NILL index, each theme encompasses multiple categories or constructs that form the main criteria of assessment within the general theme, supported by a selection of features/indicators describing the applicable features or characteristics that inform the design, construction, and operation of a nature-inspired and living laboratory building, as shown in Table 8.

In Theme 1 (Foundation and Structure), brownfield redevelopment and minimal site disturbance are given priority to guarantee that the laboratory is environmentally conscious. By using biomimicry principles to create habitat for native species, we can promote biodiversity and deepen our relationship with the natural world. Take bioswales or rain gardens, which mimic natural filtration for stormwater management, as an illustration of how the laboratory can be inspired by and integrate natural processes into its design.

In Theme 2 (Hydration and Flow), water conservation and management are emphasized. By utilizing rainwater collection, greywater reuse, and wastewater treatment to achieve net positive water use, the laboratory raises the bar for water sustainability. Biomimicry-inspired water purification systems demonstrate how the lab can benefit from understanding the efficient natural processes. Bigger water features in a range of patterns and styles help manage water and create a connection with an important element.

In Theme 3 (Energy and Power), the transition to clean energy is promoted. The laboratory can run with the least possible environmental impact thanks to net positive energy generation, which uses on-site renewable energy sources like solar or wind turbines. Biomimicry-inspired energy production technologies demonstrate how the inventiveness of nature can be used to the laboratory’s advantage. Energy-efficient laboratory equipment and HVAC systems minimize energy consumption, and evaluations of energy demand and equipment load optimize operations for long-term sustainability.

In Theme 4 (Wellbeing and Resilience), the priority is given to the happiness and well-being of the occupants. Skylights, well-placed windows, and indoor plants are examples of natural elements that can improve air quality and create a peaceful atmosphere. With less reliance on mechanical systems, natural ventilation techniques inspired by biomimicry offer a comfortable working environment. Designing with natural forms and patterns creates a space that is both harmonious and aesthetically pleasing. Furthermore, integrating the previous strategies not only satisfies buildings users and enhances their wellbeing but also ensures that laboratory buildings achieve resilience through promoting durability, flexibility, and ongoing functionality in the face of expected and unexpected challenges. As a result, laboratory buildings are capable of enduring and adjusting to diverse environmental and operational obstacles.

In Theme 5 (Skin and Breath), a strong emphasis is placed on material selection that is responsible and inclusive. By choosing low-impact, recycled, or bio-based materials over those on the LBC Red List, the laboratory reduces its environmental impact. Naturalistic design features, such as self-cleaning surfaces, can lessen the need for abrasive chemicals. Universal access features ensure that the laboratory is accessible to all, fostering equity and a sense of community. The adverse effects of waste management practices on the environment are mitigated by sustainable methods.

In Theme 6 (Balance and Harmony), a safe and co-operative atmosphere is created in the lab. Universal access features promote inclusivity, and the lab’s layout fosters collaboration and communication among researchers. Culturally sensitive design cues ensure that each researcher is treated with dignity and respect. A comprehensive safety program for laboratories prioritizes the well-being of researchers while minimizing any negative effects on the environment. Integrated safety measures, such as sufficient ventilation and emergency eyewash stations, protect researchers from potential hazards. Scientists can operate safely and effectively by funding training courses and personal safety gear.

In Theme 7 (Senses and Inspiration), the laboratory is transformed into a dynamic learning environment that stimulates the senses, spurs creativity, and advances sustainable design and biomimicry concepts. It turns into evidence of the ability of nature to serve as both an inspiration and a model for a more sustainable future.

In Theme 8 (Operation Center), the assessment index is transformed from a static building evaluation into a dynamic, living laboratory that draws inspiration from nature. This modification emphasizes the importance of continuous learning and growth. Occupant survey responses are an essential source of data for data-driven optimization of the building’s architectural features. Furthermore, life cycle assessments use rational thinking to ensure cost-effective and environmentally conscious design choices. Ultimately, the category archives design documents and guiding principles to highlight intellectual stewardship. With the help of this “living laboratory” strategy, the building itself can become a knowledge base for upcoming design projects, resulting in an ongoing cycle of learning and development.

## 5. Conclusions

This article investigated the growing prominence of nature-inspired design philosophies within the built environment, emphasizing their potential to promote sustainability and human well-being in laboratory settings. Focusing on biomimicry and biophilic design as key approaches, the paper explored how mimicking nature’s innovations and fostering connections to natural elements could benefit laboratory buildings. Additionally, the article delved into emerging methods like living building, which prioritizes user experience through co-creation and integrates sustainable and regenerative structures. By integrating these nature-inspired and living building concepts, the paper argued for the creation of dynamic and adaptable laboratory spaces that prioritized both environmental responsibility and a positive human experience. As a novel contribution to the field, this work proposed the Nature-inspired & Living Laboratory (NILL 1.0)^TM^ Building Assessment index. This index served as a guideline for the design and construction of laboratory buildings, drawing inspiration from nature and the analogy of human body systems. By embracing these nature-inspired design principles, laboratory buildings could be transformed into models for future built environments that promote a healthier and more sustainable future.

In summary, the novel assessment index that has been suggested provides a comprehensive system for evaluating the degree to which a nature-inspired and living laboratory building integrates sustainable practices, biomimicry principles, biophilic design elements, and living building features to stimulate scientific innovation and establish a comprehensive environment that encourages occupant well-being, environmental responsibility, and scientific innovation. It synchronizes the seven LBC petals with the human body system, going beyond energy and water usage, resource utilization, or material selection. The end goal is to design structures that are not only useful but also resilient, environmentally friendly, self-sustaining, regenerative, and adaptable to the surrounding environment.

Moreover, the limitations of the current work shall be highlighted. First, the current research is limited to examining human-centric approaches, such as biomimicry and biophilia, due to the increased number of laboratory facilities post COVID-19 pandemic and the developments in R&D industries, leading to an increase in the amount of time spent by laboratory users in the buildings and the need to prioritize their wellbeing and provide productive and comfortable working environments that are inspired by nature and its processes to further enhance such experiences. Consideration of nature-based and nature-positive design can definitely broaden the scope of the topic and could serve as a future expansion for the current work. To further illustrate, the Commandments of Biomimicry, similar to principles found in Cradle-to-Cradle design and Positive Development, focus on mimicking nature’s solutions to improve sustainability in human systems. Unlike net positive design frameworks, which prioritize actively increasing natural capital to counter biodiversity loss and environmental degradation, biomimicry primarily aims to optimize resource use and efficiency through bio-inspired innovations. Enhancing landscaping around buildings can improve local biodiversity and aesthetics, yet it may not adequately offset the broader impacts of biodiversity loss over the building’s lifecycle. Therefore, while biomimicry offers valuable strategies for sustainable design, integrating net positive principles would more comprehensively address the critical need to restore and regenerate natural ecosystems globally.

Furthermore, the current scope of the research mentions net positive energy, water, and waste as a critical pillar within the proposed NILL building assessment scheme. Achieving net positive energy, water, and waste reduction at the site level marks significant progress in sustainable building design. However, integrating a broader systemic perspective is essential. Addressing environmental impacts beyond immediate site boundaries is crucial for ensuring that sustainability efforts contribute effectively to overarching goals such as climate resilience, resource conservation, and ecosystem health. Therefore, expanding the scope of the current work addressing nature-inspired and living laboratory buildings to encompass systemic impacts is necessary for initiatives like the Living Building Challenge to achieve the sustainable development objectives successfully. As such, there is potential for future expansion of the current work to overcome this limitation by exploring the systems impact of designing and constructing NILL laboratory buildings.

To further solidify the NILL index’s position as a valuable tool for the design, construction, and operation of laboratory facilities, several future refinements can be explored. First, implementing a weighted scoring system would enable a more nuanced evaluation by assigning different weights to various criteria within biomimicry, biophilic design, and living building principles to allow for a more comprehensive assessment, reflecting the relative importance of each aspect in achieving the desired outcomes. Second, integrating a life cycle assessment framework could provide a more holistic evaluation by encompassing the environmental impact of materials, construction processes, and operational energy use throughout the building’s lifespan, aligning with the core principles of sustainable design. Third, the NILL index could be enhanced by allowing for regional and project-specific customization through incorporating factors like local climate, available resources, and specific laboratory functions, which could lead to more contextually relevant and achievable design goals. Finally, developing a database of NILL-rated laboratories, along with detailed case studies, would be a valuable resource for designers and building owners, as this would showcase successful implementations and best practices, inspiring future projects and demonstrating the real-world impact of the NILL index. By addressing these potential areas for improvement, the NILL index can evolve into an even more robust and practical tool, guiding the design and construction of future laboratory buildings that embrace a sustainable and human-centered approach.

## Figures and Tables

**Table 1:** Summary of major findings from the final 12 articles acquired through SCOPUS.

Title	Authors	Year	Main Findings	Theme
Biophilia in the workplace: A pilot project for a living wall using an interactive parametric design approach [14]	Assem A.; Hassan D.K.	2024	This study explored how biophilic design, specifically integrating a living wall, can enhance workplace aesthetics and well-being. It employed a parametric design approach to optimize the integration process, focusing on creating varied green wall forms inspired by natural concepts. Interactive elements were incorporated to enhance user experience and perception of the living wall’s dynamics, aiming to create a versatile ambiance conducive to diverse workplace activities. Results indicated successful implementation of these design strategies, showcasing improved user engagement and functionality within workplace environments.	Biophilic Design and Living Building
Conceptualization of Biomimicry in Engineering Context among Undergraduate and High School Students: An International Interdisciplinary Exploration [15]	Yeter I.H.; Tan V.S.Q.; Le Ferrand H.	2023	The study found that engineering students grasped the bottom-up approach of biomimetics more readily, using traditional engineering tools to apply biological knowledge for engineering solutions, while perceiving the top-down approach, which identifies technical problems and applies natural solutions, as vaguer. It suggests that combining both approaches in teaching biomimicry, along with hands-on learning, could effectively enhance student comprehension of these concepts.	Biomimicry
Short-term effects of natural view and daylight from windows on thermal perception, health, and energy-saving potential [16]	Jiang Y.; Li N.; Yongga A.; Yan W.	2022	Visual windows enhanced thermal comfort, potentially reducing HVAC energy use; physiological measures were more sensitive than subjective questionnaires in assessing their impact on occupant health, indicating positive effects on well-being by alleviating symptoms of sick building syndrome and reducing stress and fatigue indicators.	Sustainable Building and Biophilic Design
Clay 3D printing as a bio-design research tool: development of photosynthetic living building components [17]	Crawford A.; In-na P.; Caldwell G.; Armstrong R.; Bridgens B.	2022	The study utilized digital fabrication to embed living microalgae in ceramic building components, examining how design factors like geometry and firing temperature affect algae growth. It highlighted the importance of managing evaporation and moisture levels for optimal performance and proposed digital manufacturing as a method to develop viable, integrated systems for living building applications.	Living Building
Photosynthetic textile biocomposites: Using laboratory testing and digital fabrication to develop flexible living building materials [18]	Stefanova A.; In-Na P.; Caldwell G.S.; Bridgens B.; Armstrong R.	2021	The study explored the development of 3D-printed biocomposites containing the microalgae Chlorella vulgaris, which can be integrated into building materials to help sequester CO_2_, demonstrating their effectiveness in supporting algae growth despite occasional challenges in cell distribution and fluctuations. Kappa-carrageenan, full-strength BG11 nutrient medium, and Auro Clay Paint were promising when used with cotton and polyester textiles, despite occasional cell distribution challenges and fluctuations.	Living Building
The impact of a view from a window on thermal comfort, emotion, and cognitive performance [19]	Ko W.H.; Schiavon S.; Zhang H.; Graham L.T.; Brager G.; Mauss I.; Lin Y.-W.	2020	The study discovered that providing office occupants with a window view resulted in slight yet noteworthy enhancements in their thermal comfort, positive emotions, and specific cognitive functions such as working memory and concentration, in comparison to those in windowless environments. Having a window could potentially contribute to energy savings as it makes occupants more tolerant of minor thermal comfort variations. Windows were also found to boost occupants’ psychological well-being by amplifying positive emotions and minimizing negative ones.	Biophilic Design
Building on the inherent strengths of green space environments: Promoting trust, democracy, and resilience among ethnically diverse groups [20]	Hoffman A.	2020	This quasi-experimental study explored how participating in community service activities within green space environments, such as community gardens and urban forestry programs, influenced individuals’ perceptions of community service-learning programs and democratic processes associated with green space development. Sixteen volunteers shared their subjective experiences, noting increased appreciation for living things, a stronger connection with plants and animals, and a heightened sense of belonging to nature. Interviews highlighted how exposure to these environments shaped participants’ views on the importance of nature in urban settings and their personal sense of connection to both community and the natural world.	Biophilia
Leed gold but not equal: Two case study buildings [21]	Baja F.D.F.; Bajracharya S.; Freeman M.A.; Gray A.J.; Haglund B.T.; Kuipers H.R.; Opatola O.R.	2019	The study aimed to resolve issues of glare, thermal discomfort, and excessive brightness in the Education building’s west- and south-facing study spaces due to highly reflective materials. Researchers proposed an integrated shading solution with vertical fins and elongated light shelves to mitigate direct sunlight and enhance visual and thermal comfort. Additionally, the paper compared two LEED Gold-rated buildings at the University of Idaho, highlighting varying ecological performance despite similar certifications, and offered recommendations to address comfort issues in the Education building.	Sustainable Building and Biophilic Design
The R.W. Kern center as a living laboratory: Connecting campus sustainability goals with the educational mission at Hampshire college, Amherst, MA [22]	Cianfrani C.M.; Hews S.; Tor J.; Jewhurst J.J.; Shillington C.; Raymond M.	2018	The R.W. Kern Center at Hampshire College exemplified how sustainable design can educate future sustainability leaders, transforming the campus to prioritize pedestrians over cars and showcasing features like optimized building orientation, insulation, natural ventilation, and educational displays as a living laboratory for students.	Sustainable Building and Living Laboratory
Modelling to drive design: Honing the SU + RE house through performance simulations [23]	May E.	2018	Digital simulation technologies transformed architectural design by enabling direct study and manipulation of energy, water, air, heat, and sound flows impacting building occupants. The SU + RE House exemplified this advancement, showcasing how integration of data and environmental analysis techniques could create genuinely sustainable and resilient buildings. Led by Ed May from Stevens Institute of Technology and BLDGtyp, the project marked a significant shift towards designing structures that prioritize environmental performance and occupant comfort	Biophilic Design

**Table 2:** Strategies for the application of Benyus’s commandments of biomimicry to laboratory buildings.

Commandments of Biomimicry	Application to Laboratory Buildings
Use Waste as a Resource:	Design laboratories that incorporate waste-to-resource systems, utilizing lab waste for energy production or recycling materials within the facility.
Diversify and Co-operate to Fully Use the Habitat:	Create laboratory environments that mimic the diversity and co-operation found in natural ecosystems. Design spaces that accommodate various research activities and encourage collaboration among scientists.
Gather and Use Energy Efficiently:	Implement energy-efficient technologies and systems in laboratories, such as renewable energy sources, smart energy management, and energy recovery systems.
Optimize Rather Than Maximize:	Focus on optimizing laboratory processes and spaces, avoiding excessive resource use and square footage. Prioritize efficiency and functionality over unnecessary expansion.
Use Materials Sparingly:	Design laboratories with a focus on minimal material use, incorporating sustainable and low-impact materials. Prioritize durability and recyclability in material selection.
Don’t Foul Their Nests:	Ensure that laboratory activities, waste disposal, and emissions are managed in an environmentally responsible manner, minimizing negative impacts on the surrounding ecosystem.
Don’t Draw Down Resources:	Design laboratories with a commitment to sustainable resource management, avoiding the depletion of natural resources and promoting circular economy practices.
Remain in Balance with the Biosphere:	Align laboratory design with the local ecosystem, considering factors like water usage, biodiversity, and ecological balance. Implement landscaping that supports local flora and fauna.
Run on Information:	Incorporate smart technologies and information systems in laboratories for efficient data collection, analysis, and communication. Embrace data-driven decision making for sustainability.
Shop Locally:	Source laboratory materials and equipment locally whenever possible to reduce the carbon footprint associated with transportation. Support local businesses and contribute to the regional economy.

**Table 3:** Strategies for the application of biomimicry life principles to laboratory buildings.

Biomimicry Life Principles	Application to Laboratory Buildings
Evolve To Survive	Design labs to be adaptable and flexible to accommodate changing research needs, technologies, and environmental conditions. Utilize modular and adaptable infrastructure to facilitate future expansion and modifications.
Replicate Strategies that Work	Emulate proven natural designs and processes to enhance lab functionality and efficiency. Implement biophilic design elements to improve occupant well-being and productivity.
Integrate the Unexpected	Incorporate unexpected elements and unconventional approaches to foster innovation and creativity within the lab environment. Encourage cross-disciplinary collaboration and embrace serendipitous discoveries.
Reshuffle Information	Analyze and utilize data effectively to optimize lab operations, resource management, and energy consumption. Implement smart sensors and control systems to gather real-time data and make informed decisions.
Adapt To Changing Conditions	Design labs to respond to changing environmental conditions, such as fluctuating temperatures, sunlight intensity, and occupancy levels. Utilize passive design strategies to minimize energy consumption and ensure thermal comfort.
Incorporate Diversity	Create diverse lab spaces that cater to different research needs and encourage collaboration among scientists. Foster a culture of inclusivity and diversity of thought to maximize the potential of the lab environment.
Maintain Integrity through Self-Renewal	Design labs with self-healing and self-repairing mechanisms to minimize maintenance requirements and extend the lifespan of the building. Utilize renewable materials and sustainable practices to ensure long-term functionality.
Embody Resilience	Design labs to withstand natural hazards, climate change, and other disruptions. Implement robust structural systems, redundant power sources, and disaster preparedness plans to ensure continuous operation.
Be Locally Attuned and Responsive	Design labs to harmonize with the local climate, ecology, and cultural context. Utilize locally sourced materials, adapt to site conditions, and respect the surrounding environment.
Leverage Cyclic Processes	Integrate cyclical processes, such as rainwater harvesting, greywater recycling, and natural ventilation, to reduce resource consumption and minimize environmental impact.
Use Readily Available Materials and Energy	Utilize sustainable and locally sourced materials with low environmental impact. Prioritize renewable energy sources and optimize energy efficiency throughout the building.
Use Feedback Loops	Implement feedback loops to continuously monitor and improve lab performance. Utilize data analytics to identify areas for optimization and implement corrective measures.
Cultivate Cooperative Relationships	Encourage collaboration and knowledge sharing among lab users, researchers, and the surrounding community. Foster partnerships with local universities, research institutions, and businesses.
Integrate Development with Growth	Design labs that can accommodate future growth and expansion without compromising sustainability or functionality. Utilize modular and adaptable infrastructure to facilitate seamless expansion.
Self-Organize	Design labs with self-organizing capabilities, such as intelligent lighting systems and automated climate control, to optimize resource utilization and occupant comfort.
Build from the Bottom Up	Adopt a bottom-up approach to lab design, involving users, researchers, and community members in the planning and decision-making process.
Combine Modular and Nested Components	Utilize modular and nested components to create flexible and adaptable lab spaces that can be easily reconfigured and expanded as needs evolve.
Be Resource Efficient (Material and Energy)	Minimize material consumption and energy use throughout the design, construction, and operation of the lab.Implement sustainable practices and prioritize renewable energy sources.
Use Low Energy Processes	Utilize low-energy processes and technologies to minimize the environmental impact of lab operations. Employ energy-efficient equipment, appliances, and HVAC systems.
Use Multi-Functional Design	Design lab spaces with multi-functional capabilities to reduce the need for additional infrastructure and maximize resource utilization.
Recycle All Materials	Implement comprehensive recycling and waste management strategies to divert materials from landfills and promote circularity. Utilize waste streams as energy sources whenever possible.
Fit Form to Function	Design lab spaces that prioritize functionality and efficiency over unnecessary aesthetics. Follow biophilic design principles to create a harmonious and stimulating environment.
Use Life-friendly Chemistry	Implement green chemistry principles to minimize the use of hazardous chemicals and promote sustainable alternatives. Utilize nontoxic and biodegradable materials whenever possible.
Break Down Products into Benign Constituents	Design products and processes that break down into benign constituents at the end of their lifecycle, minimizing environmental impact.
Build Selectively with a Small Subset of Elements	Utilize a limited number of well-understood and sustainable materials to reduce complexity and facilitate recycling and reuse.
Do Chemistry in Water	Implement water-based chemistry whenever possible to minimize the use of harmful solvents and reduce environmental impact. Utilize water as a reaction medium and solvent.

**Table 4:** Strategies for the application of Living Building Challenge (LBC) to laboratory buildings.

Living Building Challenge		Application to Laboratories	Impact on Laboratories
Petal	Imperative		
Place	Limits to Growth Place	Design laboratories that respect ecological limits, considering local ecosystems, biodiversity, and resource availability.	Ensures the laboratory’s impact aligns with the natural capacity of the surrounding environment.
	Urban Agriculture	Incorporate green spaces and possibly rooftop gardens to promote urban agriculture within the laboratory setting.	Enhances the laboratory’s connection to nature, provides greenery for researchers, and contributes to local food production.
	Habitat Exchange	Implement measures to enhance and protect local ecosystems, possibly through partnerships with conservation organizations.	Demonstrates a commitment to preserving and enhancing the natural habitats surrounding the laboratory.
	Human-Powered Living	Promote alternative transportation methods, such as cycling or walking, and design spaces that encourage physical activity.	Aligns with a nature-inspired approach by encouraging a healthy and active lifestyle for laboratory occupants.
Water	Net Positive Water	Design water-efficient laboratories with water capture and reuse systems, ensuring a positive impact on the local water balance.	Demonstrates responsibility in water usage, mirroring natural systems’ efficiency.
Energy	Net Positive Energy	Implement renewable energy sources and energy-efficient design to achieve net-positive energy consumption.	Aligns with the sustainability aspect of a nature-inspired approach by reducing energy demand.
Health and Happiness	Civilized Environment	Design laboratories with a focus on promoting social well-being, comfort, and a sense of community.	Creates a workspace that aligns with the harmonious and civilized aspects of nature.
	Healthy Interior Environment	Prioritize air quality, lighting, and acoustics to create a healthy and comfortable indoor environment.	Ensures researchers work in spaces that support their well-being, similar to the health-promoting aspects of nature.
	Biophilic Environment	Integrate nature-inspired design elements, such as natural light, greenery, and biomimicry, to create a biophilic laboratory.	Enhances the connection between researchers and the natural world, fostering a positive and inspired work environment.
Materials	Red List	Avoid the use of materials on the Red List, prioritizing healthier and environmentally responsible choices.	Aligns with the nature-inspired principle of using materials in harmony with the environment.
	Embodied Carbon Footprint	Minimize the embodied carbon footprint of construction materials, choosing low-impact options.	Reflects a commitment to reducing the overall carbon impact, aligning with sustainable practices found in nature.
	Responsible Industry	Source materials from responsible and sustainable suppliers and promote ethical practices within the laboratory.	Demonstrates a commitment to responsible and sustainable industry practices.
	Living Economy Sourcing	Support local economies and choose materials and services that contribute to a living economy.	Aligns with the nature-inspired principle of interconnectedness and community support.
	Net Positive Waste	Minimize waste generation and implement recycling and composting systems to achieve net-positive waste.	Mirrors the efficiency and waste reduction found in natural ecosystems.
Equity	Human Scale and Humane Places	Design laboratories with a human-centric approach, focusing on comfort, accessibility, and a sense of place.	Aligns with the nature-inspired principle of creating spaces that resonate with human well-being.
	Universal Access to Nature and Place	Ensure that all laboratory occupants have access to natural elements, whether through views, green spaces, or biophilic design.	Fosters inclusivity and promotes a connection to nature for everyone in the laboratory.
	Equitable Investment	Prioritize equitable investment in laboratory facilities, ensuring fair distribution of resources and benefits.	Reflects a commitment to fairness and equity, similar to the balanced relationships in nature.
	Just Organization	Implement just and equitable policies within the laboratory organization, considering the well-being and fairness of all occupants.	Creates a work environment that aligns with the principles of justice found in nature.
Beauty	Beauty and Spirit	Design laboratories with aesthetic appeal, incorporating natural elements and inspiring spaces.	Aligns with the beauty and inspiration found in the natural world.
	Inspiration and Education	Design spaces that inspire creativity and provide educational opportunities for researchers and visitors.	Creates a laboratory environment that encourages learning and innovation, mirroring the inspiration found in nature.

**Table 5:** Comparison between the petals and imperatives of the Living Building Challenge (LBC) and the possible application within laboratory buildings.

Biomimicry Principle	Living Building Challenge	Similarities	Difference	Application to Laboratories
	Petal: Imperative			
Use Waste as a Resource	Materials: Net Positive Waste	Both emphasize minimizing waste generation and finding valuable uses for waste materials.	Biomimicry emphasizes emulating nature’s ability to transform waste into valuable resources, while the Living Building Challenge focuses on quantifying waste reduction and diversion.	Implement composting systems, establish waste segregation and recycling programs, and explore opportunities to reuse or repurpose lab materials.
Diversify and Cooperate to Fully Use the Habitat	Place: Ecology of Place	Both emphasize creating diverse and interconnected ecosystems that support a variety of life.	Biomimicry focuses on emulating nature’s complex ecosystems, while the Living Building Challenge emphasizes restoring and enhancing biodiversity on the lab site.	Integrate native landscaping, create habitats for wildlife, and promote interactions between different species within the lab’s environment.
Gather and Use Energy Efficiently	Energy: Net Positive Energy	Both emphasize reducing energy consumption and utilizing renewable energy sources.	Biomimicry focuses on emulating nature’s ability to harness energy efficiently from natural sources, while the Living Building Challenge focuses on quantifying energy production and consumption.	Implement energy-efficient appliances and lighting, optimize HVAC systems, and utilize renewable energy sources such as solar panels or geothermal systems.
Optimize Rather Than Maximize	Materials: Responsible Materials	Both emphasize using materials efficiently and responsibly.	Biomimicry focuses on emulating nature’s ability to achieve functionality with minimal material use, while the Living Building Challenge emphasizes using nontoxic, renewable, and locally sourced materials.	Select materials with low environmental impact, prioritize reusable and recyclable materials, and minimize material consumption throughout the lab’s design and construction.
Use Materials Sparingly	Materials: Red List	Both emphasize minimizing the use of harmful materials and reducing the environmental impact of materials.	Biomimicry focuses on emulating nature’s use of nontoxic and biodegradable materials, while the Living Building Challenge emphasizes avoiding red list materials and quantifying embodied carbon emissions.	Eliminate the use of hazardous materials, prioritize sustainable and bio-based alternatives, and consider the lifecycle impact of materials.
Don’t Foul Their Nests	Health & Happiness: Healthy Interior Environment	Both emphasize creating healthy and nontoxic environments.	Biomimicry focuses on emulating nature’s ability to create clean and healthy ecosystems, while the Living Building Challenge emphasizes minimizing exposure to harmful pollutants and optimizing indoor air quality.	Prioritize natural materials with low off-gassing potential, ensure adequate ventilation, and implement air filtration systems to maintain a healthy indoor environment.
Don’t Draw Down Resources	Place: Limits to Growth Place	Both emphasize living within ecological limits and respecting natural resources.	Biomimicry focuses on emulating nature’s ability to operate within resource constraints, while the Living Building Challenge emphasizes minimizing the lab’s impact on local ecosystems and resources.	Employ water-efficient fixtures, implement rainwater harvesting systems, and reduce reliance on nonrenewable resources.
Remain in Balance with the Biosphere	Place: Habitat Exchange	Both emphasize maintaining a balance with the natural world.	Biomimicry focuses on emulating nature’s ability to maintain equilibrium and resilience, while the Living Building Challenge emphasizes restoring and enhancing biodiversity.	Integrate biophilic design elements, create habitats for wildlife, and promote sustainable practices that minimize the lab’s impact on the surrounding environment.
Run on Information	Beauty: Education + Inspiration	Both emphasize the importance of knowledge and learning.	Biomimicry focuses on emulating nature’s ability to gather and process information, while the Living Building Challenge emphasizes creating a culture of learning and innovation.	Foster a culture of knowledge sharing, encourage interdisciplinary collaboration, and incorporate educational elements into the lab’s design and operation.
Shop Locally	Materials: Living Economy Sourcing	Both emphasize supporting local communities and economies.	Biomimicry focuses on emulating nature’s interconnectedness and reliance on local resources, while the Living Building Challenge emphasizes sourcing materials and equipment from local businesses.	Prioritize locally sourced materials and equipment, support local businesses, and engage with the local community throughout the lab’s development and operation.

**Table 6:** Overview of the Nature-inspired & Living Laboratory (NILL 1.0)^TM^ Building Assessment Index.

Index Theme	LBC Petal	Analogy	Connection
Foundation and Structure	Place	Skeletal and Muscular Systems	A strong foundation and musculoskeletal system provide support and stability, just as a well-designed site and building envelope are crucial for a building.
Hydration and Flow	Water	Circulatory System	The circulatory system efficiently transports water and nutrients throughout the body, similar to how a water-positive building manages and utilizes water resources.
Energy and Power	Energy	Metabolic System	The metabolic system converts food into energy, mirroring a building’s ability to generate and utilize renewable energy.
Wellbeing and Resilience	Health and Happiness	Nervous and Immune Systems	The nervous and immune systems ensure overall health and adaptation, just as a healthy building environment promotes occupant well-being and resilience.
Skin and Breath	Materials	Integumentary System (Skin) and Respiratory System	The skin protects us from the environment, and the respiratory system facilitates healthy air exchange, analogous to how a building’s materials and ventilation systems manage internal and external interactions.
Balance and Harmony	Equity	Endocrine System	The endocrine system regulates various bodily functions. Similarly, the LBC’s equity petal promotes social justice and creates a balanced and equitable environment for all.
Senses and Inspiration	Beauty and Spirit	Sensory System and Central Nervous System	The sensory system and central nervous system allow us to perceive and experience the world, similar to how a building’s aesthetics and functionality can inspire and uplift its occupants.
Operation Center	Integration of all petals	Human Brain	The human body integrates all its different systems that are controlled by the brain, while the LBC relies on the collective results of all its petals to control its overall performance and become a regenerative self-sustained building.

**Table 7:** Alignment between the NILL index main themes, the relevant LBC petal, and the Biomimicry Life Principles.

Index Theme	LBC Petal	Aligned Biomimicry Principles
Foundation and Structure	Focuses on minimizing site disturbance, restoring ecological functions, and integrating biomimicry strategies for sustainable site management.	Reshuffle Information Build from the Bottom Up Combine Modular and Nested Components
Hydration and Flow	Emphasizes achieving net positive water use through biomimicry-inspired water management strategies.	Leverage Cyclic Processes Use Feedback Loops
Energy and Power	Focuses on achieving net positive energy through biomimicry-inspired renewable energy generation.	Use Low Energy Processes Leverage Cyclic Processes Integrate the Unexpected
Wellbeing and Resilience	Integrates biophilic design principles inspired by nature to enhance occupant well-being and connection with the environment, while also fostering a beautiful and inspiring workspace.	Embody Resilience (variation and decentralization) Maintain Integrity through Self-Renewal Cultivate Co-operative Relationships
Skin and Breath	Prioritizes minimizing environmental impact through sustainable material selection, biomimicry-inspired design features, and ensuring an accessible and inclusive environment for all users.	Recycle All Materials Use Multi-Functional Design Fit Form to Function Break Down Products into Benign Constituents Build Selectively with a Small Subset of Elements Do Chemistry in Water
Balance and Harmony	Ensures the laboratory fosters a sense of community, collaboration, and a safe and healthy work environment.	Incorporate Diversity Cultivate Co-operative Relationships
Senses and Inspiration	Combines biophilic design elements that appeal to the senses with biomimicry-inspired solutions to foster creativity, innovation, and a sense of connection with nature, and integrating the Living Lab concept to design the laboratory as a research platform for biomimicry and sustainable technologies, actively collecting and sharing data to advance the field.	Reshuffle Information Use Feedback Loops Self-Organize Build from the Bottom Up Combine Modular and Nested Components
Operation Center	Brains and building control centers are both information hubs. While the brain processes senses and commands to control the body, the laboratory operation center analyzes sensor data (ex. temperature) to adjust lab systems for optimal performance.	Replicate Strategies that Work Adapt To Changing Conditions Use Feedback Loops Be Locally Attuned and Responsive Use Readily Available Materials and Energy Be Resource Efficient (Material and Energy)

**Table 8:** The main themes, dimension, and indicators of the Nature-inspired & Living Laboratory (NILL 1.0)^TM^ Building Assessment Index.

Theme	Dimension	Indicators	Description	Reference
Foundation & Structure (FD)	Site & Location (SL)	Sustainable Site Selection & Development	Minimal site disturbance with priority to refurbishment of old sites, brownfield redevelopment prioritization, or location near ancient site.	[31,32]
		Building Orientation	Well-oriented building and an envelope that is tightly sealed prevent unwanted heat transfer	[33]
		Habitat Creation	Rooftops, pollinator gardens, or green walls as habitats for native species mimicking natural ecosystems.	[31,32]
		Proximity to Public Transit	Availability of nearby bus station; main road access.	[34,35]
		Pedestrian Area	Designated walking area with minimal vehicular access.	[31]
	Nature-inspired Structures (NIS)	Nature-inspired Facades	Reactive facades resembling the mechanism of pinecone in responding to environmental stimuli (open and close); integrated solar panels into exterior façade resembling the lotus flower’s ability to absorb sunlight efficiently or integrated photobioreactors in building façade with microalgae (to reduce thermal loads with the absorption of radiation)	[36,37,38]
		Nature-inspired Surrounding	Use of artwork showcasing biomimicry examples.	[39]
		Green Space Coverage	Maximize green space coverage ratio and promote plant canopies for shading and sheltering. Prioritize the use of native species to enhance biodiversity or use plants that provide shading.	[4,40]
		Access to Nature	Provide balconies, terraces, courtyards, or roof slopes for direct connection to nature.	[31,41]
Hydration & Flow (HF)	Water Sourcing (WS)	Waste Water Treatment System	Greywater treatment housed on the grounds or local system affiliated with central treatment plant; can be inspired by mussel filtration.	[42,43]
		Seawater Desalination	Seawater desalination system housed on the grounds or local system affiliated with central desalination plant.	[31,44]
		Rain Water Capturing	Rainwater collection system.	[31,42,43]
		Dew & Condensate Capturing	Dew harvesting or condensation capture.	
		Nature-based Water Systems	Biofiltration systems based on plants’ inherent ability to filter air or bioswales; wetlands or rain gardens mimicking natural filtration for stormwater management.	[45]
	Water Management (WM)	Water Use Optimization	Closed-loop systems in nature.	[46,47]
		Water Efficient Practices	Low-flow fixtures and water-saving technologies (foot pedals) in laboratory sinks and equipment.	[48]
		Treated Wastewater Repurposing	Using treated wastewater for toilet flushing.	[42,43]
		Treated Grey Water Repurposing	Greywater reuse for irrigation.	
Energy & Power (EP)	Energy Generation (EG)	Renewable Energy	Solar or PV panels (mimicking photosynthesis) or wind turbines (inspired by bird wings) to generate net positive energy.	[49,50]
		Excess Energy Sharing	Excess renewable energy shared with the local grid.	[51,52]
		Passive Heating/Cooling	Natural ventilation or solar chimneys	[4,36]
	Energy Management (EM)	Equipment Load	Energy-efficient equipment selection and placement for minimized energy use throughout the lab’s lifespan	[48,53]
		Energy Demand Optimization	Demand-controlled ventilation (DCV) systems, LED lights, and automatic light control.	[36,54,55,56]
		Energy-efficient HVAC System	Heat recovery ventilation (HRV) or energy recovery ventilation (ERV) systems mimicking natural ventilation processes, or integrating PV panels with HVAC Systems.	[36,57]
		Energy-efficient Cold Storage	Optimization of cold storage units, including insulation levels and practices for reducing unnecessary cooling cycles.	[58,59]
		Energy-efficient Laboratory Equipment	Low-power microscopes, fume hoods with occupancy sensors, and autoclaves with heat recovery systems with certifications like ENERGY STAR or equivalent.	[60,61,62]
		Equipment Utilization	Practices like scheduling, shared use protocols, and right-sizing equipment for specific needs; equipment auction or swapping.	[48]
		Equipment Management	Smart Energy Management Systems or software and practices for regular maintenance and calibration of equipment to ensure optimal performance and efficiency.	[36,63,64]
		Energy Conservation	Energy conservation measures like charged batteries for equipment.	[43,63]
Wellbeing & Resilience (WR)	Biophilic Design & Sensory Engagement (BDSE)	Visualizing Nature & Water	Maximize windows (floor-to-ceiling) with views of nature and calming water features.	[19,41,65,66,67]
		Natural light	Natural light patterns through strategically placed skylights and operable windows.	[31,68]
		Natural Sounds	Introduce natural sounds (e.g., water fountains or waterfalls and birdsong recordings).	[69,70,71]
		Quiet zones	Create designated quiet zones for focused work.	
		Indoor planting	Utilize indoor plants with air-purifying properties and aromatic benefits (e.g., lavender and rosemary).	
		Sustainable Cleaning	Avoid harsh chemical odors through sustainable cleaning practices and material selection.	[72,73,74]
		Natural Materials	Incorporate natural materials like wood and stone into the design for a connection with the natural world.	[75,76]
		Heating System	Floor heating system to maintain a constant laboratory temperature.	[49]
	Restorative Environment (RE)	Nature-inspired Ventilation	Ventilation inspiration by termite mounds or equivalent/alternative innovative approach.	[77,78]
		Natural Forms & Patterns	Incorporation of natural forms and patterns for a harmonious space and features promoting human amusement and the rooting of culture, spirit, and place	[31]
		Shading Solution	Maximize the direct exposure of the solar rays to the glazing facades in the cooling season and minimize the same gains in the heating season.	[36]
		Sustainable Materials & Finishes	Low-VOC paints, sealants, and adhesives to minimize indoor air pollution.	[79,80]
Skin & Breath (SB)	Materials & Assets (MA)	Building Material	Use of thermal mass materials that passively absorb and radiate heat	[81]
		Building Envelope	Lightweight building envelope structures with good insulation, high light penetration, and diffusion.	[36,82]
		Prohibited Materials	Avoidance of materials on the LBC Red List.	[31]
		Low-impact Material	Durable and comfortable furniture (e.g., bamboo), mushroom-based packaging for lab supplies, and materials with little maintenance.	[31]
		Recycled Material	Durable and reusable labware made from recycled materials whenever possible.	[83,84]
		Biobased Material	Self-healing material that heals and patch up small cracks or composite binders (including waterproof and frost-resistant gypsum binders).	[85,86]
		Locally-sourced material	Use locally sourced wood, stone, or other natural building materials	[31]
		Nature-inspired Asset Design	Design of self-cleaning surfaces inspired by lotus leaves to minimize harsh chemicals.	[87]
	Waste Management (WM)	Internal Waste Segregation	Segregation of plastics, glass, paper, biohazard waste, chemical waste, laboratory wastewater, and grey water.	[48,88,89]
		External Waste Segregation	Segregation to landfills or treatment facilities.	
		Waste Recycling	Clean, repackage, and reuse non-sharp equipment. Recycling/composting bins or membership in recycling programs.	[48]
		Waste Minimization	Replace single-use plastics with sustainable alternatives like metal loops and reusable wooden sticks or use of bio-based consumables where possible.	[90]
		Waste Bioremediation	Utilize plants or microbes to break down pollutants in soil or water.	[91,92]
		Hazard Reduction	Use of less hazardous solvents whenever possible, prioritizing options with lower toxicity and environmental impact and solvent recycling.	[93,94]
Balance & Harmony (BH)	Occupant & Public Engagement (OPE)	Universal Access Features	Accessible doors and smooth ramps for elderly and special needs.	[31,36]
		Interactive Laboratory Layout	Open floor plan with designated collaborative zones.	[31]
		Culturally inclusive Design Elements and Amenities	Prayer and Meditation Spaces, Lactation Rooms and Nurseries, General Food Court, Washrooms for users with special needs and elderly people, and Multilingual Signage/systems.	[31,68,95]
		Public Education & Awareness	Develop educational programs and tours for occupants and the public to showcase the lab’s sustainable features and biomimicry inspiration and integrate biomimicry and sustainability education into the research priorities.	[31,96,97]
	Safety & Security (SS)	Occupant Safety Programs	Implementation of a comprehensive laboratory safety program that prioritizes the well-being of lab users and minimizes environmental impact.	[98,99]
		Building Safety Features	Proper ventilation systems for fume hoods, emergency eyewash stations, and clear signage for hazardous materials in the building. Gas and fire detectors.	[31,100,101,102]
		Occupant Safety Measures	Personal Protective Equipment (PPE)	[103,104]
		Building Security Measures	Presence of surveillance camera and controlled door access; emergency exits.	[31]
		Continuous Building Operation	Presence of back-up energy generators.	[105]
Senses & Inspiration (SI)	Living Laboratory (LL)	Real-Time Dashboards	Integrate real-time dashboards showcasing the lab’s environmental performance, including energy use, water consumption, waste generation through a design inspired by biomimicry’s focus on feedback loops and biophilic design’s connection with nature.	[106,107,108]
		Living Wall Research Platform	Design a living wall or vertical garden that serves as a research platform for studying plant-based air purification technologies and their effectiveness in laboratory settings.	[36]
		Data Collection & Sharing	Comprehensive data collection system to monitor energy use (motion sensors), water consumption, waste generation, and indoor air quality.	[106,109]
	Adaptive & Evolving Design	Growth & Expansion	Modular Construction or equivalent/alternative innovative approach.	[110,111,112]
		Reconfiguration & Enhancement	Universal Lab Shell with adaptable Mechanical, Electrical, and Plumbing (MEP) or equivalent/alternative innovative approach.	[113,114]
		Building Innovation	Emphasis on innovative design, construction, and operational practices that push the boundaries of sustainability in living laboratories, considering potential for advancements in energy-efficient laboratory equipment or closed-loop waste management systems within research activities (open category)	[115]
Operation Center (OC)	Feedback Loop (FL)	Occupant Perception	Regular surveys to measure occupant awareness of building features and their significance.	[116,117]
		User Satisfaction	Regular surveys to measure level of satisfaction	[118]
	Logical Reasoning (LR)	Material Selection Criterion	Life Cycle Assessment (LCA): to evaluate the environmental impact of the bio-inspired materials or processes throughout their life cycle, considering factors like energy consumption, material extraction, and end-of-life disposal.	[119,120]
		Asset Selection Criterion	Feasibility Study (FS) and/or Cost–Benefit Analysis (CBA)	[121,122]
		Awareness & Preparedness	Risk Assessment (RA) and Contingency plans	[123,124]
	Knowledge & Intellect Treasury (KIT)	Conceptual Record Preservation	Preservation of design plans, blueprints, and log records of construction process, including origins of nature-inspired ideas.	[125]
		Data Preservation	Back-up servers for databases and archive of printed records	[126,127]

## Data Availability

Data are contained within the article.
